# Generation of a Stable Transgenic Swine Model Expressing a Porcine Histone 2B-eGFP Fusion Protein for Cell Tracking and Chromosome Dynamics Studies

**DOI:** 10.1371/journal.pone.0169242

**Published:** 2017-01-12

**Authors:** Renan B. Sper, Sehwon Koh, Xia Zhang, Sean Simpson, Bruce Collins, Jeff Sommer, Robert M. Petters, Ignacio Caballero, Jeff L. Platt, Jorge A. Piedrahita

**Affiliations:** 1 Comparative Medicine Institute, North Carolina State University, Raleigh, North Carolina, United States of America; 2 Department of Molecular Biomedical Sciences, College of Veterinary Medicine, North Carolina State University, Raleigh, North Carolina, United States of America; 3 Department of Surgery and Microbiology and Immunology, University of Michigan Health System, Ann Arbor, Michigan, United States of America; 4 Department of Animal Science, College of Agriculture and Life Sciences, North Carolina State University, Raleigh, North Carolina, United States of America; Utah State University, UNITED STATES

## Abstract

Transgenic pigs have become an attractive research model in the field of translational research, regenerative medicine, and stem cell therapy due to their anatomic, genetic and physiological similarities with humans. The development of fluorescent proteins as molecular tags has allowed investigators to track cell migration and engraftment levels after transplantation. Here we describe the development of two transgenic pig models via SCNT expressing a fusion protein composed of eGFP and porcine Histone 2B (pH2B). This fusion protein is targeted to the nucleosomes resulting a nuclear/chromatin eGFP signal. The first model (I) was generated via random insertion of pH2B-eGFP driven by the CAG promoter (chicken beta actin promoter and rabbit Globin poly A; pCAG-pH2B-eGFP) and protected by human interferon-β matrix attachment regions (MARs). Despite the consistent, high, and ubiquitous expression of the fusion protein pH2B-eGFP in all tissues analyzed, two independently generated Model I transgenic lines developed neurodegenerative symptoms including Wallerian degeneration between 3–5 months of age, requiring euthanasia. A second transgenic model (II) was developed via CRISPR-Cas9 mediated homology-directed repair (HDR) of IRES-pH2B-eGFP into the endogenous β-actin (ACTB) locus. Model II transgenic animals showed ubiquitous expression of pH2B-eGFP on all tissues analyzed. Unlike the pCAG-pH2B-eGFP/MAR line, all Model II animals were healthy and multiple pregnancies have been established with progeny showing the expected Mendelian ratio for the transmission of the pH2B-eGFP. Expression of pH2B-eGFP was used to examine the timing of the maternal to zygotic transition after IVF, and to examine chromosome segregation of SCNT embryos. To our knowledge this is the first viable transgenic pig model with chromatin-associated eGFP allowing both cell tracking and the study of chromatin dynamics in a large animal model.

## Introduction

Pigs are an attractive research model in the field of surgical and procedure training, disease progression and pathology, translational research, and regenerative medicine/stem cell therapy, due to their anatomic, genetic, and physiological similarities with humans. In addition, the availability of the pig genome sequence, the development of somatic cells nuclear transfer (SCNT) and high efficiency genome editing tools such as Transcription activator-like effector nucleases (TALENs) and Clustered regularly interspaced short palindromic repeats (CRISPR-Cas9), have allowed precise and efficient genetic engineering in the pig [1–5].

With available transgenic pig technology and the generation of more suitable biomedical research models for translational research, the need for more effective methods for in vivo and ex-vivo cell tracking have increased. This is particularly true in the area of regenerative medicine. While cells can be loaded ex-vivo with a range of dyes, these dies disappear over time limiting their usefulness for long-term studies [6–8]. Therefore, the development of fluorescence proteins as molecular tags has allowed complex biochemical processes to be correlated with protein functionality in living cells [6]. In addition, genetic engineering of encoded biological fluorescent proteins have marked an evolution in the field of stem cell biology, allowing the development of cell-traceable systems and the ability to track the fate of adult stem cells for therapeutic purpose in biomedical models [9, 10]. Among these molecular tags, the most widely used one is the green fluorescent protein (GFP) from the jellyfish *Aequorea victoria* [6, 11]. The mutant variant, eGFP (enhanced), has been shown to be more stable and to fold correctly at 37°C, and can be fused with virtually any protein of interest allowing co-localization of eGFP and the protein of interest. [6].

Based on this concept, transgenic mice, rats, rabbits and pigs expressing eGFP under a variety of conditions have demonstrated their usefulness in basic and translational research [7, 12, 13]. Furthermore, nuclear fluorescence tagging, by adding nuclear localization signals that translocate eGFP into the nucleus [14], or by fusing eGFP to proteins that bind chromatin, has allowed the tagging of the nucleus versus the cytoplasm and a better understanding of the complexity of cell cycle/division, chromosome abnormalities associated with cancer biology, and real-time chromosome dynamics [15]. One of the approaches used to accomplish this is via fusion of eGFP to the histone 2B protein (H2B) [16, 17]. Histone H2B is a one of the main core proteins of the nucleosome and plays a key role in chromatic assembly [18]. By fusing eGFP to H2B it is possible to co-localize the eGFP to the chromosomes and observe their behavior in real time. While transgenic mice models expressing the fusion protein H2B-eGFP under different conditions have been described [19], to our knowledge the development of a stable transgenic pig expressing such fusion protein is currently lacking. Such model can be used as a tool in multiple fields of biomedical research including cell tracking after transplantation, cell fusion analysis, cell division kinetic analysis in vivo and in vitro [20], and chromatin remodeling in early embryos [21].

Here we describe two transgenic pig models expressing pH2B-eGFP. One developed using random insertion and a well-characterized, strong, ubiquitous promoter (pCAG) protected by Scaffold/Matrix attachment regions (MARs). MARs are DNA elements that are part of the nuclear matrix, and are known to reduce what is a known as "position effect variegation" (PEV). PEV is characterized by heterogeneous expression (and in some cases silencing) of randomly integrated transgenes [22–24]. The use of MARs has been described in multiple species including swine [25, 26]. The second model generated via CRISPR-Cas9-mediated HDR insertion of pH2B-eGFP transgene in the ACTB locus, allowing its expression to be regulated by the ACTB promoter.

## Material and Methods

### Animal Welfare

This study was carried out in strict accordance with the recommendations in the Guide for the Care and Use of Laboratory Animals of the National Institutes of Health. The animals used in this study were obtained from a university owned herd, and all animal procedures were approved by the Institutional Animal Care and Use Committee of North Carolina State University (Raleigh, NC). Animals were sacrificed by one of two methods, intravenous injection of sodium pentobarbital, or penetrating captive bolt euthanasia followed by jugular exsanguination. Both methods meet the recommended guidelines of the American Veterinary Medical Association for euthanasia in pigs. All surgeries were performed under isoflurane anesthesia, and a post surgical regimen of bupivacaine, Banamine-S was administered to minimize pain.

### Model Development

#### Model I (pCAG-pH2B-eGFP/MAR); non-targeted integration

**Fig 1:**
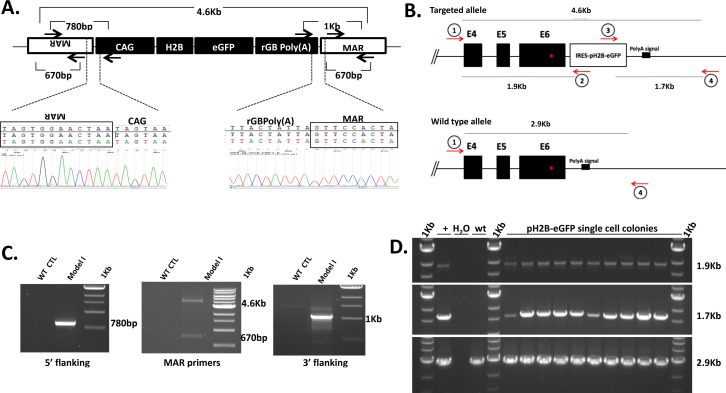
Transgenic approaches for generation of Model I and Model II pigs. (A) Representative genomic organization of Model I insert from line 1 showing structure of the pH2B-eGFP construct and the location of the MARs. Overall organization was confirmed by sequencing. (B) Schematic representation of the structure of the wild-type and targeted ACTB locus. Diagnostic PCR amplicons and location of primers are shown. (C) PCR amplicons of from control (WT CTL) and Model I genomic DNA confirming the overall organization of the insert. Amplicons were sequenced to confirm identity. (D) Analysis of pH2B-eGFP positive colonies confirming insertion of the pH2B-eGFP into the ACTB locus. Amplicons were sequenced to confirm identity.

Model I line was generated by random integration in the genome and used a strong, well-characterized promoter and MARs to control PEV of the pH2B-eGFP. To generate Model I, three constructs were used (Fig 1A and 1C), PGK-puro, human interferon-β MARs and pCAG-pH2B-eGFP-rabbit Globin poly A. The porcine H2B coding region (single exon) was amplified from the pig genome via PCR, sequenced, fused to eGFP and cloned into a pCAG expression vector (Clontech, catalog #6085–1). The 812 bp human interferon-β MAR was amplified (and sequenced confirmed) from pCpGfree-vitroNLacZ (InvivoGen, Catalog# pcpgvtn-lz) plasmid using the forward and reverse primers, 5’-CTGCAGGAATTCAGTCAATATGTTC-3’ and 5’-GCCACAGATGTTACTTAGCCTTT-3’. Finally, PGK-puro was used for selection.

Two independent porcine fetal fibroblast (pFFs) lines isolated from two different fetuses were used for generation of Model I. All pFFs lines were obtained by breeding Landrace X Yorkshire mix boars and sows. Male wild type pFFs (400,000 cells) were mixed with 2 μg linearized pCAG-pH2B-eGFP plasmid, 2 μg hIFNβ/MAR and 500 ng PGK-Puro plasmid, and electroporated at 490 V, 3 pulses of 1 ms duration using the BTX Electro-cell manipulator ECM2001. Electroporated cells were plated in alpha MEM supplemented with 15% FBS and penicillin/streptomycin. Twenty-four hours later, cells were placed on selection media containing 0.75 mg/ml puromycin. Following selection for 10 days, surviving colonies were expanded and briefly analyzed by epi-fluorescence microscopy to phenotypically evaluate colonies based on nuclear pH2B-eGFP expression. Colonies showing heterogeneous pH2B-eGFP expression were discarded. Colonies that showed homogeneous and ubiquitous nuclear pH2B-eGFP expression were further expanded and tested to confirm the presence of MARs before using for SCNT. Two independent Model I lines where established and studied separately. The presence of MARs adjacent the pH2B-eGFP was examined by PCR as shown in Fig 1A. Primers used were MAR forward 5'- ATG AGA TGT GTG GGG ATA GAC AGT GA-3'and MAR reverse 5'- CTTTACTGGGGTGTTGCAAATATTTTCTGTC-3', reverse pCAG 5'- ACTATGGGAACATACGTCATTATTGAC-3' and forward rGBPoly(A) 5'-TGAGCATCTGACTTCTGGCTAA-3'. All PCR reaction were performed under the following conditions 98°C for 1 min, 35 X (98°C for 10 s, 62°C for 10 s, 72°C for 60 s), 72°C for 1 min, hold at 4°C.

**Fig 2:**
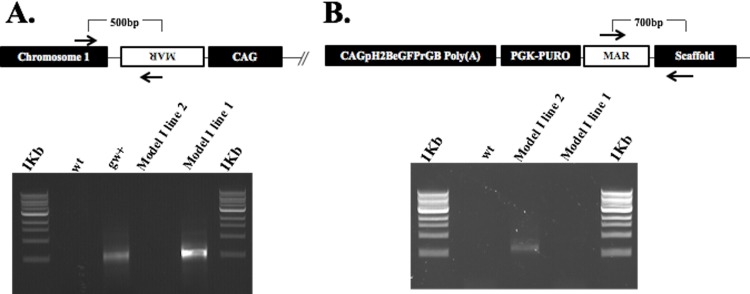
Confirmation of independent insertion sites in both Model I lines. (A) Using the sequence obtained from the GenomeWalking system, internal primers were designed to the predicted insertion site and MAR flanking region, and genomic DNA from both lines and control wild type amplified by PCR. Correct amplicons were only detected in line 1 DNA confirming the GenomeWalker predicted insertion site. (B) Similarly, using the predicted organization of the transgene and the genomic flanking region PCR primers were designed and wild type, line 1 and line 2 genomic DNA amplified. As expected, only line 2 DNA yielded the correct amplicon. Both (A) and (B) results prove that the transgene from both lines used to generate model I have different organizations and are inserted in different genomic locations. Arrows indicate location of PCR primer sites.

In order to identify the transgene integration site the Universal GenomeWalker 2.0 kit was used (Clontech, cat#636405). Adapter-ligated libraries were generated with genomic DNA digested with four different blunt-end restriction endonucleases (DraI, EcoRV are showed) and CAG-pH2B-eGFP specific primers were designed to work in conjunction with adaptor primers (provided with the kit). A positive specific band from line 1 EcoRV digested/adaptor ligated library (1.4Kb) was obtained by PCR using a custom designed CAG reverse and adaptor primers. The band was gel extracted and submitted for DNA sequencing revealing 900 bp sequence with 100% homology to the pig genomic region identified that allowed mapping and confirmation by PCR of the insertion site (Fig 2).

The insertion site for the additional Model I line was identified by whole genome sequencing by using a commercial service (Rapid Genomics, Gainsville). For library preparation the DNA sample was quantified using Quant-iT PicoGreen dsDNA Assay Kit (Life Technologies, Carlsbad) and normalized to 15 ng/ul. A total of 500 ng of DNA was physically fragmented to an average fragment size of 400 bp. An Illumina-compatible DNA library was constructed by end-repairing the sheared DNA, A-tailing and adapter ligation. The ligated libraries were PCR-amplified for 9 cycles. The product was sequenced on one lane of Illumina HiSeq X-Ten (Illumina, San Diego) with paired end reads of 150 bp each. Paired end raw reads were cleaned and trimmed with trim galore/0.4.1 (Babraham Bioinformatics—Trim Galore! Babraham Bioinformatics—Trim Galore!. [http://www.bioinformatics.babraham.ac.uk/projects/trim_galore/]. Reads where one pair mapped to the transgene and its mate pair mapped to the pig genome were used to identify possible insertion sites. To identify these pairs, bowtie2 [27] was used to map the cleaned reads to the transgene sequences. The bowtie mapping results were filtered with bamsamba [28] for reads where only one mate mapped to the transgene. Blast [29] was used to map the mate of the read that hit the transgene to the pig genome (Genome assembly: Sscrofa10.2, http://useast.ensembl.org/Sus_scrofa/Info/Index).

#### Model II (ACTB-IRES-pH2B-eGFP); homology directed repair (HDR)

Model II was developed using CRISPR-Cas9 and HDR to drive integration of the pH2B-eGFP into one allele of the ACTB locus resulting in ubiquitous expression of the pH2B-eGFP. The CRISPR-Cas9 system consisted of the bi-cistronic expression vector px330 expressing Cas9 and sgRNA under U6 promoter (addgene, catalog # 42230). An sgRNA target sequence to the 3' UTR of the ACTB locus was identified using ZiFit Targeter Version 4.2 (http://zifit.partners.org/ZiFiT/ChoiceMenu.aspx). This sequence was added to the plasmid using complimentary oligos, 5'-CACCGACGGTGAAGGTGACAGCAGT-3' and 5'-AAACACTGCTGTCACCTTCACCGTC-3’.

For the generation of the IRES-pH2B-eGFP targeting construct, the internal ribosomal entry site (IRES) sequence from Encephalomyocarditis virus was amplified from pCAGIG plasmid (addgene, cat# 11159), and fused to pH2B-eGFP from the pCAG-pH2B-eGFP plasmid. Porcine ACTB homology arms of 1Kb were amplified from pig genomic DNA and cloned into the targeting vector flanking IRES-pH2B-eGFP (Fig 1B). The targeting vector did not contain the β-actin CRISPR-Cas9 target sequence. The strategy consisted of generating a chimeric mRNA of ACTB and IRES-pH2B-eGFP by inserting the transgene in the 3' untranslated region (UTR). The stop codon and the endogenous ACTB Poly(A) signal was kept intact.

For electrochemical transfection, 5x10^5^ wild type male pFFs were transfected using the Amaxa Nucleofector kit (U-23 settings) (Lonza,Verviers, Belgium). Transfection groups included pMAXGFP (500ng) as positive control, non-transfected pFFs as negative control, and ACTB CRISPR-Cas9 (500ng) plus circular targeting vector (500ng) as the HDR group. Ninety-six hours post transfection cells were trypsinized, resuspended in fetal fibroblast culture media (alpha MEM supplemented with 15% FBS and penicillin/streptomycin) and Fluorescence Activated Cell Sorted (FACS). Wild type pFFs served as negative control and pFFs transfected with pMAXGFP plasmid as a positive control. Cells were briefly trypsinized, washed twice in PBS and sorted for GFP on a Beckman Coulter MoFlo Legacy system (sort pressure 25PSI using a 100 micron nozzle) using a 488nm laser for side-scattered light (SSC) and forward-scattered light (FSC), and a 530/40 filter to capture the eGFP emission. Positive cells from HDR group were harvested and plated at low density (100 cells/100mm petri dish) for single cell colony expansion. Five to seven days later, single cell colonies were picked and transferred to a 24-well plate.

To confirm the targeted insertion of IRES-pH2B-eGFP into the ACTB locus, primers were designed to amplify a 1.9kb 5' boundary fragment and a 1.7kb 3' boundary fragment. As shown in Fig 1B, primers were designed to specifically amplify IRES-pH2B-eGFP flanked by the correct location of the ACTB 3'UTR region. For 5' boundary, primers were 5' CTGAGTCTCCTTTGGAACTCTGCAG 3' and 5' CTTGTTGAATACGCTTGAGGAGAGC 3'. PCR conditions were 98°C for 1 min, 35 X (98°C for 10 s, 66°C for 10 s, 72°C for 30 s), 72°C for 1 min, hold at 4°C. For the 3' boundary primers were 5' GTACAACTACAACAGCCACAACGTC 3' and 5' CTGACAGTTCCATTTCTCACGTTCC 3' under following condition 98°C for 1 min, 35 X (98°C for 10 s, 68°C for 10 s, 72°C for 30 s), 72°C for 1 min, hold at 4°C. For determining homozygous vs. heterozygous HDR, primers were designed to bind outside the areas of homology arms of ACTB, resulting in amplification of a 2.9kb amplicon for unmodified wild type allele. Primers were 5' CTGAGTCTCCTTTGGAACTCTGCAG 3' and 5' CTGACAGTTCCATTTCTCACGTTCC 3' under condition of 98°C for 1 min, 35 X (98°C for 10 s, 68°C for 10 s, 72°C for 45 s), 72°C for 1 min, hold at 4°C.

### Somatic Cell Nuclear Transfer

Both models (I and II) were generated using the same SCNT protocol [30]. All chemicals were purchased from Sigma (St. Louis, MO) unless otherwise specified. Oocytes from mixed commercial breed sows were collected from local slaughterhouses. Cumulus cells were removed from the oocyte by vortex for 5 min in 0.1% bovine testicular hyaluronidase. Oocytes were incubated in manipulation media (Ca-free NCSU-23 with 5% FBS) containing 5 μg/mL bisbenzimide and 7.5 μg/mL cytochalasin B for 5 min. Following the incubation period, oocytes were enucleated by removing the first polar body and metaphase II plate. Single cells expressing pH2B-eGFP were injected and fused to each enucleated oocyte. Fusion/Activation was induced by two DC pulses of 140V for 40 μsec in 280 mM mannitol, 0.001 mM CaCl_2_, and 0.05 mM MgCl_2_. After fusion/activation, oocytes were placed back in NCSU-23 medium with 0.4% BSA and cultured at 38.5°C, 5% CO_2_ in a humidified atmosphere for less than an hour, before being surgically transferred into a synchronized recipient.

### Histology and Immunohistochemistry (IHC)

To determine the usefulness of the pH2B-eGFP marker, frozen and paraffin embedded samples were examined for pH2B-eGFP expression after sectioning without use of anti-GFP antibody (direct GFP detection) as well as after using anti-GFP antibody at 1/400 dilution (Alexa Fluor 555 conjugated, rabbit polyclonal IgG (indirect detention, cat#A-31851, ThermoFisher). Five to 10 μm sections were obtained from both paraffin-embedded and OCT-embedded tissues. For paraffin embedded blocks samples were fixed in 10% neutral buffered formalin overnight, followed by fixation with 70% ethanol and paraffin embedding. For OCT-embedded tissue blocks samples were fixed with 4% paraformaldehyde overnight followed by 30% sucrose solution (also overnight). Once fixed, samples were embedded in OCT and frozen in liquid nitrogen. The cryosectioned tissues slides were immunostained as described below. Slides were permeabilized with 0.25% Triton X-100 in PBST (PBS with 0.1% Tween 20) for 10 min. Slides were incubated for one hour at room temperature in 10% BSA in PBST, then with Alexa555 conjugated-Anti-GFP (Invitrogen) overnight at 40°C. Next day, slides were washed three times with PBST and mounted with VECTASHIELD Mounting Medium with DAPI (Vector Laboratories) and visualized with TE2000 fluorescence microscope (Nikon, Melville, NY, USA). The same slides used for immunostaining and/or other slides from serial sections were subsequently used for hematoxylin and eosion (H&E) staining as previously described [31].

For natural eGFP fluorescence analysis, tissues from both Model I and Model II were fixed with 4% (wt/vol) paraformaldehyde (PFA) in PBS overnight, followed by 30% sucrose solution and OCT embedding. Cryosection from multiple organs were counterstained with 1 mg/mL of DAPI in Vectashield mounting medium. Primary cultured stem cells were analyzed for eGFP expression without fixation. All samples were observed with a fluorescence microscope (LSM510 Meta, Zeiss, Thornwood, NY).

In addition, bone marrow mesenchymal stem cells (BM-MSCs), umbilical cord mesenchymal stem cells (UC-MSCs), neural stem cells (NSC), and chondrocytes were isolated from both models and examined for expression of pH2B-eGFP. For isolation of BM-MSCs, bone marrow was collected in PBS supplemented with 5% FBS and Streptomycin, Penicillin, and Amphotericin. The bone marrow was manually fragmented using sterile forceps, followed by manual agitation to release the cells and filtered using a 40μM filter. The cell suspension was gently laid on a Fycoll gradient and centrifuged at 400 × g for 40 minutes to enrich for mononuclear cells. Mononuclear cells were plated overnight on DMEM supplemented with 10% FBS and Streptomycin/Penicillin to enrich for adherent cells. The following day non-adherent cells were removed by washing with PBS 2x, and remaining adherent cells isolated by tripsinizisation. The cells were further characterized by RT-PCR to confirm expression of the MSC markers CD105, CD90 and lack of expression for CD34.

UC-MSCs were isolated as described previously [32]. Briefly, pig umbilical cords were aseptically collected from newborn piglets and umbilical arteries and vein removed. The remaining tissue, containing the Wharton jelly, was minced in DMEM media with Penicillin, Streptomycin and Amphotericin B (Corning, Inc. NY). The explants were transferred to six-well plates containing the above media along with 20% fetal bovine serum (FBS) and left undisturbed for 5–7 days to allow migration of cells from the explants. They were fed thereafter twice weekly and passaged as necessary (cells passaged at 80–90% confluence). The cells were further characterized by RT-PCR to confirm expression of the MSC markers CD105, CD90 and lack of expression for CD34.

The techniques used for cell isolation of pig fetal NPCs used in this study were adapted and modified from a protocol described previously for humans [33]. Fetal brains collected from D42 fetuses were enzymatically digested with collagenase type IV 0.5mg/ml (Worthington, Lakewood, NJ) and 0.05% trypsin (Corning, Inc, NY) for 20 minutes at 37°C, and the resulting cell suspension was washed, filtered through a 40 uM filter and plated in ultra low attachment plates (Corning, Inc. NY) for the development of neurospheres. The cell culture media consisted of Dulbecco’s modified Eagle’s medium/F-12 with high glucose (Corning, Inc. NY) containing L-glutamine (200 mM), BIT9500 (10% by volume; StemCell Technologies, Vancouver, British Columbia, Canada), EGF (20 ng/ml), bFGF (40 ng/ml), platelet-derived growth factor–AB (20 ng/ml), and antibiotics. Neurospheres were visualized after 5 and 6 days and images taken. The cells were further characterized by RT-PCR and shown positive for the neural stem cell markers GFAP, TUJ1 and negative for CD34.

To isolate primary chondrocytes costal cartilage was minced into fine fragments (less than 1 mm^2^), fragments washed in Dulbecco’s phosphate buffered saline (DPBS; Cellgro, Manassas, VA) and digested with 1.2% type II collagenase (Worthington, Lakewood, NJ) at 37°C for 6 hr. The cell suspension was filtered through a cell strainers (70 μm; BD Falcon, Franklin Lakes, NJ) to obtain single cells. Cells were washed three times in DPBS and re-suspended in DMEM containing 10% FBS. The number of total cells and viable cells were calculated by Trypan blue staining (Sigma, St. Louis, MO) and by counting with a Bright-Line^TM^ Hemacytometer (Sigma; average cell yield: 470,000 cells per gram). Viable cells were plated into 6- and 24-well plastic culture dishes at a density of 15,000 cells/cm^2^. Cells were cultured at 37°C under 5% O_2_ conditions in DMEM (Corning, Inc. NY) with 10% FBS. Identity of chondrocytes was confirmed by expression of COL2 and ACAN.

### pH2B-eGFP Expression After In Vitro Fertilization (IVF) and SCNT

To examine pH2B-eGFP expression in the early IVF embryos, frozen epididymal sperm from an pH2B-eGFP boar was used for IVF as previously described [34]. Matured oocytes with a first polar body were washed three times in manipulation media (Ca-free NCSU-23 with 5% FBS). Approximately 30 to 35 oocytes were transferred into 50 μL droplets of in vitro fertilization (IVF) medium covered with mineral oil that had been equilibrated at 38.5°C in 5% CO2 in air. A 0.1 mL frozen semen pellet was thawed at 38.5°C in 10 mL of sperm-washing medium. After washing twice by centrifugation (1,900RPM at room temperature, 4 min), cryopreserved epididimal spermatozoa were resuspended with fertilization medium to a concentration of 1 × 10^6^ cells/mL. Fifty microliters of the sperm sample was added to the fertilization droplets containing the oocytes, giving a final sperm concentration of 0.5 × 10^6^ cells/mL. Oocytes were co-incubated with sperm for 4 to 6 h. After fertilization, oocytes were washed three times and cultured in 500 μL in NCSU-23 medium with 0.4% BSA in 4-well dishes at 38.5°C in 5% CO_2_ for 6 days. Expression of pH2B-eGFP was evaluated through preimplantation development, from fertilization until blastocyst stage.

For SCNT embryos, following fusion and activation, embryos were fixed with 4% (wt/vol) paraformaldehyde (PFA) in PBS for 30 min at room temperature, counterstained with 1 mg/mL of DAPI in Vectashield mounting medium (Vector Laboratories, Burlingame, CA) to stain DNA and mounted on glass slides to examine pH2B-eGFP expression. In addition, unfixed embryos were used for time-lapse imaging following SCNT activation and until the 2–4 cells stage.

For live imaging, IVF or SCNT embryos were transferred to drops of medium on a glass-bottomed dish (20 embryos per 50μl drop), placed in the incubator on the microscope stage and incubated at 38.5°C under 5% CO_2_ in air. The system has a Z motor and an auto X–Y stage. Images were acquired in two channels (light microscope, green fluorescence) using the auto shutter. Device control and image analysis were performed using MetaMorph software (Molecular Devices, Sunnyvale, CA, USA). For time-lapse observations, images were taken over 50 h at 15 min intervals.

### Expression Levels of pH2B-eGFP in Model I and Model II

Expression levels were compared using Western blotting, flow cytometry fluorescence intensity profiles, and image analysis of fluorescent intensities in two cell types (BM-MSC and chondrocytes). Cells previously isolated and frozen from two aged-matched animals were used for each Model I and Model II donors and a wild type control. Passage 3 cells were thawed, plated (5 x10^5^/well) in 100mm dishes and grown to 100% confluence. Once confluent, total proteins were extracted and 20 μg of total protein separated on a 4–20% precast stain-free gel (Bio-Rad, cat#456–8094). Proteins were transferred to a PVDF membrane, and the membrane probed with rabbit IgG anti-GFP polyclonal antibody (1:5000; Abcam, cat#ab6556) at 5°C overnight, followed by incubation at room temperature for 1 hour with goat anti-rabbit IgG HRP-conjugated (1:10000; Abcam, cat#ab97051). The PVDF membrane was then washed and treated with ECL for detection and image capture. For β-Actin detection the membrane was stripped with 0.5M Tris HCL, 10% SDS and β-mercaptoethanol at 50°C for 45 minutes and checked to ensure loss of pH2B-eGFP signal. It was then probed with rabbit IgG anti-β actin polyclonal antibody (1:5000, Abcam, cat#ab8227) at 5°C overnight, followed by incubation at room temperature for 1 hour with goat anti-rabbit IgG HRP-conjugated (1:10000; Abcam, cat#ab97051). PVDF was washed and treated with ECL for detection. The predicted size for pH2B-eGFP fusion protein was 41kDa, and β-actin 43kDa. Intensity profiles were obtained using Image Lab (Bio-Rad) and normalized to β-actin.

Model I and Model II eGFP fluorescence intensity was also compared using cultured BM-MSCs and chondrocytes from two animals per model. All cell lines used were in passage 3. BM-MSCs and chondrocytes cells were plated in triplicate 35mm wells (2 x10^5^/well) and grown to 100% confluence in pFF media. Five microscopic fields (40X) were randomly selected from each well, a gate/active region created to represent the cell nucleus corresponding to pH2B-eGFP signal, and GFP intensity was acquired for each cell nucleus using the active gate. The GFP intensity profile from 100 cells/field was measured with the active gate (500 cells per replication, 1500 per animal). GFP intensity analysis was performed using MetaMorph software (Molecular Devices, Sunnyvale, CA, USA). The same strategy was applied to wild type cells and gate used to establish background intensity, and values used as threshold. Statistical analysis comparison was performed using one-tailed student's t-test with a significance value of *p* < 0.05. In addition, flow cytometry was used to compare fluorescence intensity between Model I and Model II BM-MSC cells. Prior to flow cytometry analysis, cells from control, Model I and Model II donors were thawed, washed twice with PBS, plated at an equal density (50.000 viable cells per 100mm plate) and allowed to growth to 80% confluence. Cells were then tripsinized, washed twice with PBS, and analyzed by flow cytometry for eGFP mean fluorescence intensity using a LRSII system (488nm laser and 530/40 filter). At least 400,000 cells per sample were examined.

## Results

### Generation and Analysis of Model I Pigs

Two independent pFF lines, selected as described in material and methods, were used as nuclear donors for SCNT. Reconstructed SCNT embryos (181 and 126; S1 Table) were transferred into two surrogate gilts generating thirteen piglets from one line and seven piglets from the other (Model I parental generation, P1). Pigs from both Model I lines developed neurological clinical signs between three to five months of age (hind limb lameness, generalized muscular tremor, ataxia, incoordination) and had to be humanely euthanized. To determine whether the effect was SCNT-related, cauda epididymal sperm were isolated from a Model I boar, frozen and used for *in vitro* fertilization (IVF) with wild type oocytes obtained from the slaughterhouse. A total of 212 zygotes obtained via IVF were transferred to a recipient sow, resulting in the birth of three piglets, with two piglets expressing pH2B-eGFP (Model I, F1 generation). Model I F1 piglets developed similar neurological clinical signs as the P1 generation animals and were humanely euthanized at five months of age for further necropsy evaluation.

While the two Model I lines were made independently, we examined the insertion sites to ensure that the effect seen was not due to an insertional inactivation. Using the GenomeWalker Universal Kit (Clontech, Mountainview, CA) a 900 bp flanking the insertion site was identified in one of the two Model I lines. Mapping of this region indicated that the transgene was located in intron 2 of the adhesion G protein-coupled receptor G6 gene (ADGRG6) located in chromosome 1 (http://useast.ensembl.org/Sus_scrofa/Location/View?db=core;r=1:25368175-25427711;tl=l8Jow023VfD7h62x-1889388-483094307). In spite of multiple attempts, the GenomeWalker system did not yield any positive results with the second Model I line. To identify the insertion site in this line, the whole genome was sequenced as described previously. Results identified the overall organization of the insert as well as the genomic flanking region. The insertion site was located in an as yet unmapped region of the pig genome. This region is defined as GL896235 (Scaffold ChrUScaf4119, NW_003541011.1). As shown in Fig 2 the accuracy of both the GenomeWalker and the whole genome sequencing results were confirmed by PCR. For the Model I line 1, internal primers were designed to chromosome 1 (5'-ATGGCCTTTATGGTGTTGATGTAG-3') and MAR (5'-AGCTCAGAAACAGACCCATTGATATA-3') to amplify a 500 bp amplicon. For Model I line 2 primers were designed to Scaffold GL896235 (5'-CCTCCCTCAGGTGACAGTGAG-3'), and a MAR primer was used as forward primer (5'-AGCTCAGAAACAGACCCATTGATATA-3'), to amplify a 700 bp amplicon. Additional sequence and mapping data is provided in S1 File. The results confirm that both lines are independent and support that the Model I phenotype observed is not due to the location of the transgene.

### Generation and Analysis of Model II Pigs

To generate Model II transgenic pigs, pFFs were co-transfected with custom designed ACTB CRISPR-Cas9 and a pH2B-eGFP targeting vector (Fig 1B) and analyzed for targeted integration of the pH2B-eGFP into the ACTB locus (Fig 1D). GFP positive cells were isolated via FACs analysis revealing a HDR efficiency of 2.4% (S1 Fig). Sorted eGFP-positive cells were used to generate clonally derived colonies and analyzed for DNA targeted integration via PCR. Ten independent pFFs colonies were selected for PCR analysis, all being positive and heterozygous for IRES-pH2B-eGFP integration into the ACTB locus (Fig 1D). Positive cells from one colony were used for SCNT and reconstructed embryos transferred into surrogate gilts. Three transgenic boars (Model II parental generation) were generated from one recipient after transferring 119 embryos. Two Model II parental boars are currently over 28 months old and present no abnormalities. The third boar died prior to weaning of undetermined causes. Heterozygous Model II boars were used to artificially inseminate three wild type Landrace sows, resulting in three litters. Two litters were carried to term with a total of thirteen pH2B-eGFP positive and eight negative piglets, and one pregnancy was collected at day 42 for isolation of primary cells. Transgene transmission rate was 55.8%, in concordance with Mendel's Law upon Chi Square test (*p* = 0.05). Unlike Model I animals, no deleterious phenotype has been detected at 28 months in any of the Model II P1 or F1 (12 months) transgenic animals.

### pH2B-eGFP Expression From Model I and Model II Transgenic Pigs

**Fig 3:**
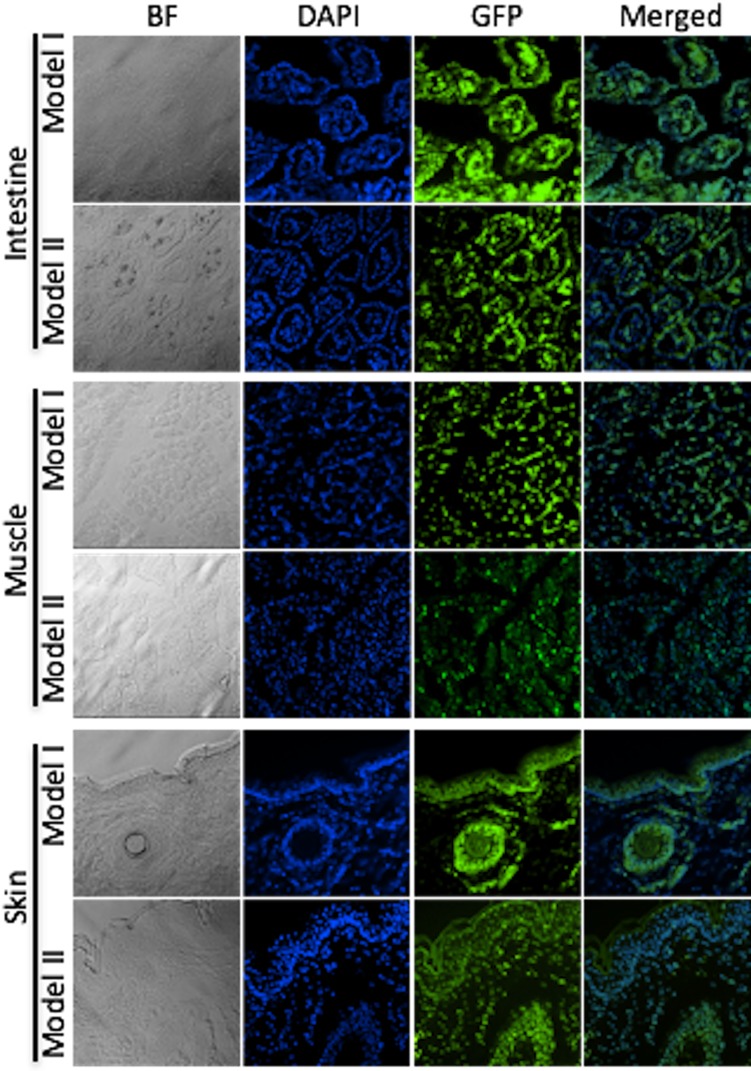
Ubiquitous pH2B-eGFP expression in Model I and Model II pigs. Fluorescence microscopy images from OCT frozen sections from intestines, skeletal muscle, and skin are show for 40X magnification for bright field, DAPI, GFP and merged GFP+DAPI. Images indicate ubiquitous expression of nuclear GFP.

**Fig 4:**
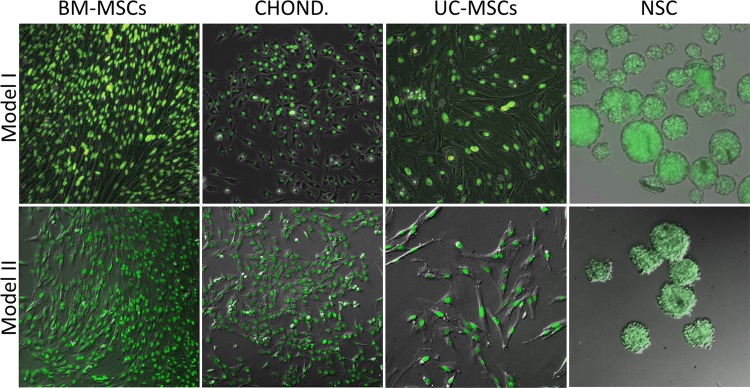
Expression of pH2B-eFGP in *in vitro* cultured adult stem/progenitor cells isolated from Model I and Model II pigs. Fluorescence microscopy (10X) showing nuclear specific expression of GFP detected from bone marrow mesenchymal stem cells (BM-MSCs), chondrocytes, umbilical cord mesenchymal stem cells (UC-MSCs) and neural stem cells (NSCs) derived from Model I and Model II pigs; pH2B-eGFP was ubiquitously expressed and nuclear localized.

**Fig 5:**
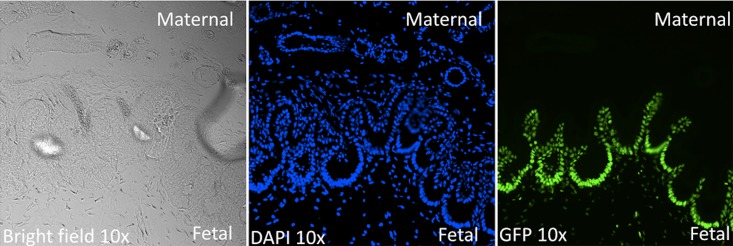
pH2B-eGFP expression from Model II 42 day fetal chorionic membrane. Fluorescence microscopy of OCT embedded fetal-maternal chorionic interphase at 42 days of gestation, showing 10x magnification for bright field, DAPI, GFP and DAPI + GFP. pH2B-eGFP expression is confined to the fetal side of fetal membrane.

**Fig 6:**
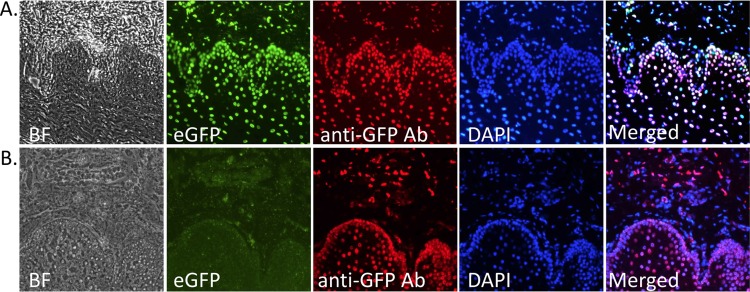
Immunohistochemical analysis of nuclear-specific eGFP expression in OCT embedded vs. paraffin embedded tongue samples. Nuclear-specific eGFP expression was confirmed through direct detection of eGFP fluorescence or by the use of Alexa-Fluor555 conjugated anti-GFP antibody staining on OCT embedded tongue (top row images). The natural GFP fluorescence signal was absent from paraffin-embedded samples, but detection of GFP could be accomplished via immunohistochemistry with Alexa-Fluor555 conjugated anti-GFP antibody. This indicates that the procedures used for processing paraffin embedded samples abolished the natural eGFP fluorescent signal but this can be recovered by the use of anti-GFP antibodies.

To determine if pH2B-eGFP expression was ubiquitous and non-variegated, multiple tissues from Model I and Model II piglets (P1 and F1 generation) were collected and OCT embedded cryo-sections analyzed via fluorescence microscopy. Nucleus-specific eGFP expression was co-localized with DAPI staining in intestine, skeletal muscle, and skin (Fig 3). We further confirmed nucleus-specific eGFP expression in the heart, brain, lung, bladder and kidney (data not shown). Primary cultures of stem/progenitor cells were also established from various tissues of newborn piglets or D42 fetuses, including BM-MSCs, UC-MSCs, fetal neural stem cells (NSCs) and chondrocytes (Fig 4). Expression of pH2B-eGFP with intranuclear localization of GFP was demonstrated in all cells types examined. In both Model I and Model II samples, eGFP expression was ubiquitous and localized to the nucleus. No variegated expression could be detected in either model. In addition, in pregnancies carrying pH2B-eGFP expressing fetuses, the fetal-maternal placental interphase (endometrium/placental) was easily distinguished by eGFP versus DAPI fluorescence microscopy evaluation at 42 days of gestation (Fig 5). To determine the effect of fixation on pH2B-eGFP detection, OCT and paraffin embedded samples were compared. As shown in Fig 6, the fixation protocol used in paraffin embedded samples squelches the fluorescent pH2B-eGFP signal. However, the protein can still be detected by the use of anti-GFP antibodies.

**Fig 7:**
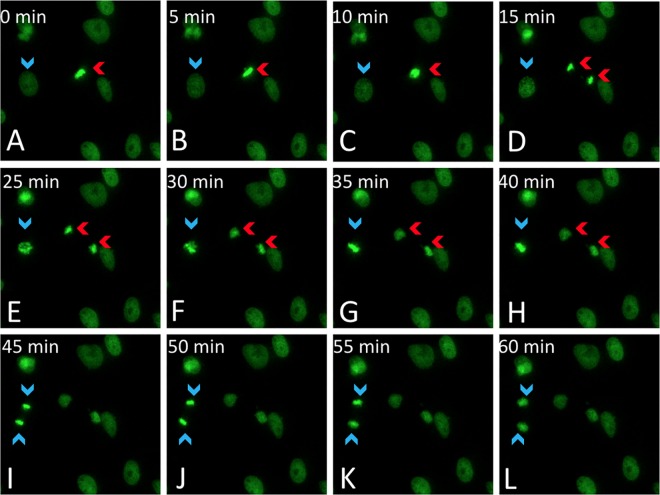
Live imaging of mitosis of Model II (ACTB-IRES-pH2B-eGFP) porcine fetal fibroblasts. Time-lapse GFP fluorescence images of cultured Model II pFFs taken at 5 min intervals. Red arrows indicate cell progression from metaphase to cytokinesis (A through H). A cell progressing from prophase to telophase is shown by blue arrows (D through L). The clear and intense chromosome-associated signal allows visualization of chromosome movements in dividing cells in real time. It also allows a rapid assessment of cell cycle synchronization protocols.

To demonstrate the usefulness of the pH2B-eGFP to examine nuclear dynamics during mitosis, pH2B-eGFP pFFs were cultured *in vitro* and analyzed via live imaging fluorescence microscopy (Fig 7). We found that chromatin positioning through pH2B-eGFP microscopy visualization allowed staging of the cell cycle and a clear visualization of the metaphase plate using conventional (versus confocal) photomicroscopy.

### pH2B-eGFP Expression From IVF and SCNT Embryos

**Fig 8:**
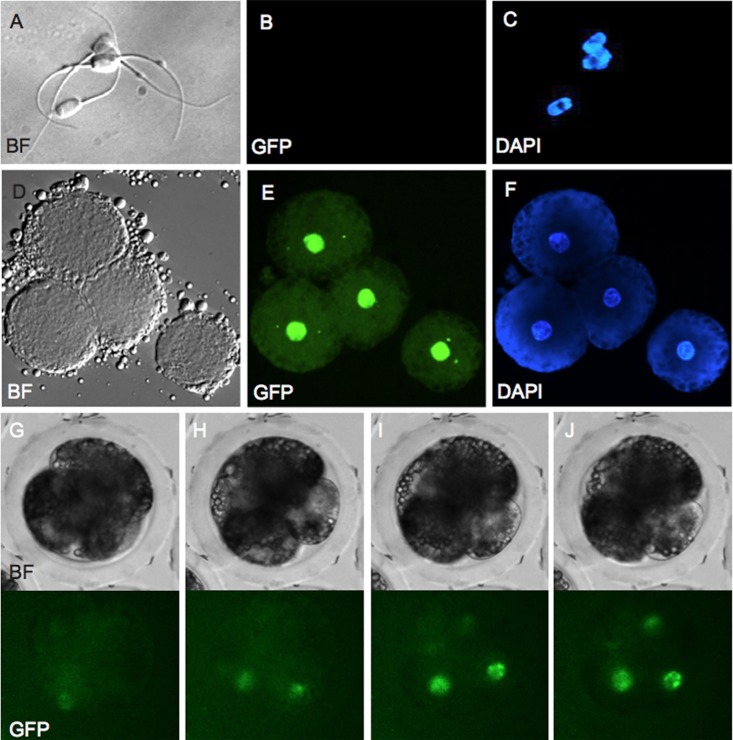
Zygote genome activation in IVF embryos. Sperm from an pH2B-eGFP boar was used to in vitro fertilize wild type oocytes and the appearance of the pH2B-eGFP signal detected using time lapse (every 5 min) photography. Both the oocyte and sperm are pH2B-eGFP negative at fertilization as expected. Nuclear GFP is observed only after transcription and translation from the sperm DNA, thus modeling the maternal to zygotic transition. A representative picture of spermatozoa is shown in (A) bright field, (B) GFP and (C) DAPI. Reactivated expression of pH2B-eGFP is shown by co-localization of (D) bright field, (E) GFP and (E) DAPI from late 4-cell stage embryo. Representative time-lapse images of reactivation of pH2B-eGFP during the 4-cell stage are shown at (G) 54.5, (H) 55, (I) 55.5 and (J) 56 hr post-fertilization in bright fields (top) and GFP (bottom).

**Fig 9:**
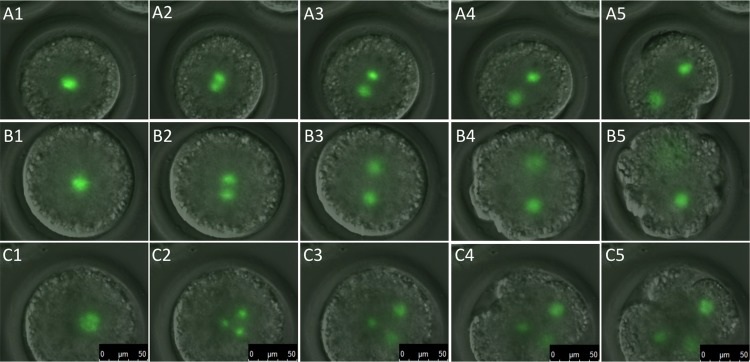
Normal and abnormal chromosome segregation in SCNT embryos generated from pH2B-eGFP donor cells. Time-lapse images (bright field and GFP merged) obtained during the first cleavage division. A. Images (left to right) show first cell division with normal cleavage, spindle formation and chromosome segregation through pH2B-eGFP visualization. B. Images shows chromosome scattering (B5) and zygote fragmentation in one of the two nuclei resulting from the first zygotic division. C. Images show multi-polar spindle formation resulting in abnormal zygote chromosome segregation and cleavage.

Nuclear transfer and IVF was performed with Model I and II donors. The generated embryos were observed either using live-cell imaging or collected, fixed and stained with DAPI at various stages after SCNT or after IVF. For IVF generated embryos, the nuclear eGFP signal could be detected starting at 68–69 hr after embryos were removed from the IVF drop (Fig 8), indicating transcription and translation of the pH2B-eGFP and modeling the maternal to zygotic transition in pigs (4 to 8 cells) [35]. For SCNT embryos, the pH2B-eGFP signal was evident from the moment the embryos were reconstructed (fusion) until the blastocysts stage, allowing visualization in real time of chromosome segregation during blastomere cleavage. Fig 9 shows representative images of normal and abnormal chromosome segregation and cleavage patterns after SCNT.

### Model I versus II pH2B-eGFP Fluorescence Intensity

**Fig 10:**
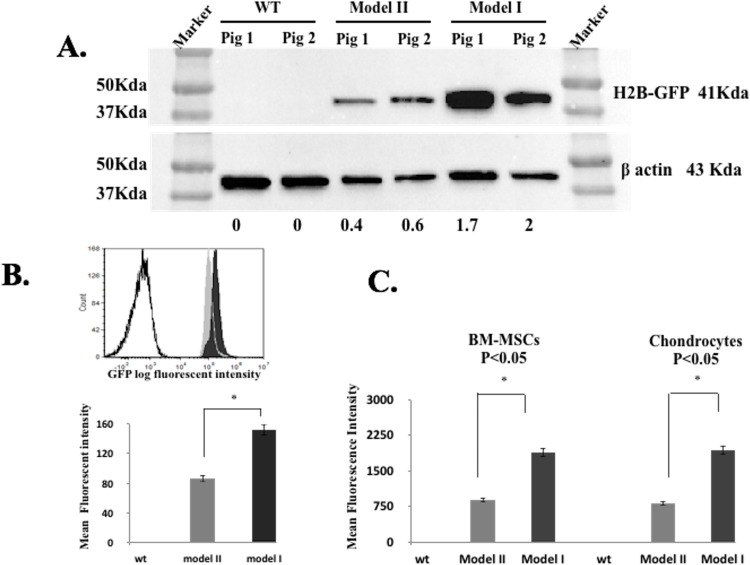
Comparison of pH2B-eGFP expression between Model I and Model II pigs. **(A)** Western blot for pH2B-eGFP and β-actin from BM-MSC isolated from Model I, Model II and controls (N = 2). When normalized to ACTB, expression levels of H2B-GFP were 1.7 and 2.0 for Model I and 0.4 and 0.6 for Model II indicating a greater than 1 fold difference between the two models. (B) Flow cytometry analysis of wild type, Model I and Model II BM-MSCs. Overlay histogram data from a single samples and a graph showing mean values from triplicates samples are shown. As for the Western data, Model I expression was approximately 2X greater (150.8 vs.86.6) than Model II expression (*p* < 0.05). (C) Microscopy fluorescence intensity comparison between passage 3 BM-MSCs and chondrocytes from Model I and Model II pigs. Image analysis of fluorescent signal was analyzed as described in Materials and methods. From each cell type the intensity of nuclear signal in 1500 cells was measured (three replicates, 500 cells per replicate). Similar to the other two methods of analysis, this method shows an approximate 2X greater signal intensity between Model I and Model II and this difference was conserved in both cell types.

When examining the OTC frozen sections we noticed a subjective difference in the intensity of the pH2B-eGFP signal between Model I and II tissues. This was confirmed by measuring protein expression by Western analysis. As shown in Fig 10, BM-MSC from Model I animals had more than twice the level of pH2B-eGFP. In addition, two different cell types; BM-MSCs and chondrocytes were examined by fluorescent microscopy. Signal intensity measurements supported the Western results with both cell types showing a significant increase in pH2B-eGFP signal intensity in Model I compared to Model II (Fig 10B). Finally, this difference was confirmed by flow cytometry. Model I and Model II BM-MSCs were analyzed via flow cytometry for mean GFP fluorescence. Approximately 400.000 passage 2 BM-MSC from each phenotype were tested in triplicate, with wild type cells serving as negative control. Mean GFP fluorescence for Model I (pH2B-eGFP/PGK/MAR) and Model II (β actin IRES-pH2B-eGFP) were 151.8x10^3^ and 87.6x10^3^, respectively (Fig 10). All three methods yielded analogous results with a significant (*p* < 0.05) higher level of expression or signal intensity in Model I versus Model II samples/animals.

### Wallerian Degeneration Accompanied by Muscle Fiber Atrophy in Model I Transgenic Pigs

**Fig 11:**
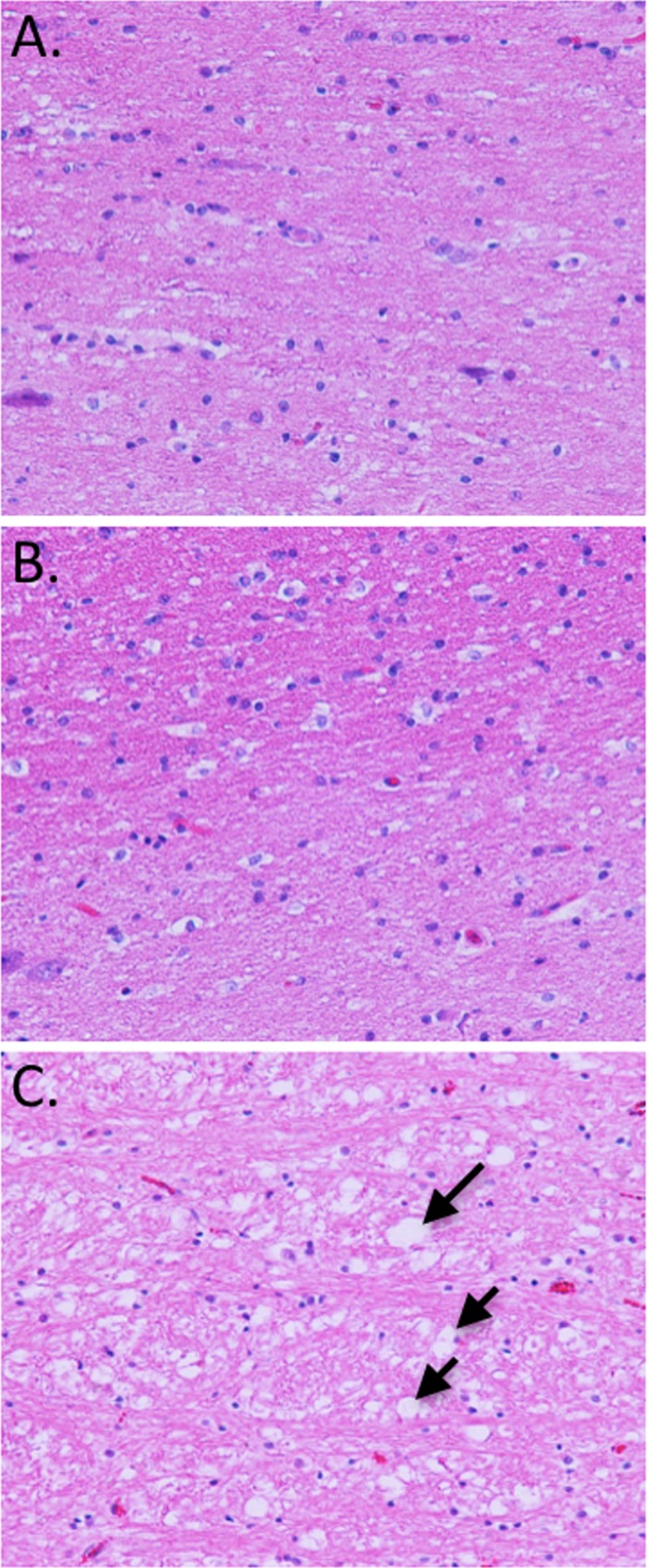
Wallerian degeneration from white matter of the Brain from Model I pig. H&E microscopic images (40X) of brainstem region (white matter) for six months old wild type pig (A), 6 months Model II pig (B) and five months old Model I pig (time when neurological signs were severe). Digestion chambers are indicated by black arrows, revealing loss of normal tissue architecture. Few vessels contain aggregates of red cells and fibrin. None of this abnormalities were present in wild type or Model II pigs.

As mentioned previously, all Model I P1 and F1 pigs developed neurological clinical signs with onset around three to five months of age. In all Model I animals, signs were consistent, with initial hind limb lameness, followed by hind limb ataxia and tremors, leading to progressive loss of hind muscle control, at which point animals were humanely euthanized. To determine the probable cause of the neurological symptoms, tissues from an affected F1 generation pig euthanized at five months were submitted for pathological evaluation. Microscopic histopathology findings included mild to moderate multifocal Wallerian degeneration of multiple regions of the brain and spinal cord (Fig 11) as well as multifocal skeletal myofiber polyphasic degeneration and atrophy. None of these lesions were observed in age-matched Model II pigs or in wild type controls (Fig 11). In addition, macroscopic necropsy findings included generalized coagulopathy with fibrin deposition in abdominal cavity, pericardium sac and pulmonary thromboembolism. Minimal necrosis or inflammation was identified. In contrast to Model I, none of the Model II P1 or F1 animals have shown similar neurological signs at 28 months of age for the P1 generation and 12 months for the F1 generation.

## Discussion

Transgenic pigs harboring and expressing green fluorescent proteins under different conditions have been described [36–43]. However, identification and quantification of engrafted donor cells after cell/tissue transplantation remains challenging due to strong auto-fluorescence, especially when GFP is expressed in the cytoplasm [44, 45]. In addition, the diversity of cell phenotype and shapes make it difficult to distinguish/count GFP-positive donor cells when utilizing automated systems. This difficulty can be overcome via nuclear GFP labeling, allowing easy and convenient cell tracking after stem cells/tissue transplantation studies. Nuclear localization of GFP can be achieved by addition of a nuclear localization signal peptide [46] or by fusion of GFP with proteins of the nucleosome core such as histones (i.e. H2B). H2B-GFP expression in cell lines [16] or transgenic mouse models [17] have been described and shown to be of great value in the field of stem cell tracking, cancer biology and chromosome dynamic studies [47, 48].

Here we describe two pH2B-eGFP transgenic pig models expressing chromatin associated GFP. Model I made via random integration of pCAG-pH2B-eGFP and MARs, and Model II via CRISPR-Cas 9-mediated integration of IRES-pH2B-eGFP into the ACTB locus. The ACTB locus was chosen as it is known to be expressed in all tissues and has been used previously to drive exogenous genes [49, 50]. Another commonly used ubiquitously expressed locus is the ROSA26 locus. This has been widely used in mice and more recently in swine [51–54]. However, there have been reports that ROSA26 expression can vary widely in particular in certain cell types. For instance Cutler and colleagues [51] reported that expression levels of lacZ from the ROSA26-lacZ reporter mouse changed drastically during remodeling of arteries. Of greater concern for transplantation studies is the discrepancy between ROSA26 locus expression and other markers. Theise and colleagues [55] reported that after bone marrow transplantation of ROSA26-lacZ cells into irradiated mice, splenic engraftment was 90% when measured by Y-chromosome analysis but only 50% when measured by lacZ staining. This suggests that under certain conditions the ROSA26 locus will give inaccurate information of the degree of engraftment after transplantation.

Overall, our data demonstrates that both Model I and Model II expressed pH2B-eGFP in a ubiquitous manner in all cells examined. Consistent high-level expression in all nuclei and no evidence of position effect variegation were seen in either model. Moreover, the nuclear signal can be easily visualized without the use of antibodies in frozen OTC sections in all tissues tested (Figs 3 and 4). By combining GFP detection with DAPI staining, the number of transgenic nuclei in a given field of vision can be easily calculated. This greatly facilitates quantitation of engrafted cells after transplantation, which can be more difficult to do when using cytoplasmic GFP due to auto fluorescence and the complex 3D structure of cytoplasmic membranes. In addition, if higher resolution tissue architecture is required, the pH2B-eGFP protein can be detected using widely available anti-GFP antibodies in more harshly fixed tissues where the endogenous GFP signal is lost (Fig 6). Similarly, the use of the pH2B-GFP marker allows clear differentiation between the maternal and the fetal interface during pregnancy and this facilitate studies examining the potential interactions and trafficking of cells between the two compartments in normal and diseased placentas (Fig 5).

Histone tagged fusion fluorescence protein has been suggested as a strategy to label chromosomes in living cells and animals and has been applied to the study of chromosome and cell cycle dynamics [56–60]. When performing live image fluorescence microscopy of Model I and Model II fetal fibroblasts, we could visualize chromatin position and dynamics through pH2B-eGFP signal during various stages of cell cycle division (Fig 7). This finding was in concordance with the previous work with mouse cells expressing H2B-GFP [16, 19]. Cell cycle progression and chromosome dynamics can be evaluated with this model for a variety of stem or somatic differentiated cells, supporting that the developed pig model will be useful in field of chromosome dynamics and cancer biology. We could also examine the maternal to zygotic transition (MTZ) after IVF by looking at the timing of appearance of chromatin-associated pH2B-eGFP and confirm previous observations that it occurs during the early stages of the 4-cell stage [61].

During SCNT the donor somatic nucleus undergoes a series of complex modifications characterized by reprogramming events initiated by maternal factors. Remodeling, not of the underlying genetic sequences that comprise the genome, but of the epigenome, is a key factor controlling differentiation and development at this stage. However, in most, if not all, mammalian species used for SCNT to date there is a large proportion of reconstructed embryos that are lost at the very early cleavage stages. Examinations of both centrosomes and microtubules have shown that there is a number of defects associated with abnormal chromosome segregation and abnormal cytokinesis [62–67]. In the case of the pig, these defects are exacerbated the longer the time period between fusion and activation [67]. However, in all of the manuscripts referenced above, examination of nucleosome dynamics and microtubules required fixation of the zygotes. Unlike cytoplasmic GFP, H2B-eGFP is embedded in the incoming donor cell chromatin as part of the nucleosomes and as such is protected from degradation. If no additional H2B-GFP is made, the signal intensity is lowered by half with each cell division. This property has been used to examine the proliferative state of certain stem cells in vivo [20] and allowed us to examine chromosome dynamics in SCNT embryos prior to the maternal to zygotic transition. While our IVF data indicates that newly synthetized pH2B-GFP is detectable by the 4-cells stage, there is still sufficient pH2B-GFP in the incoming donor cells to allow careful examination of chromosome segregation in early cleavage stage SCNT embryos (Fig 9). This allowed real time visualization of chromosome dynamics and rapid identification of multi-polar spindle formation (Fig 9). This provides a unique tool not only to better understand the dynamics of chromosome segregation in SCNT embryos but equally important to rapidly evaluate potential treatments to reduce these defects-that can affect as many as 50% of SCNT embryos [66].

However, while both Model I and II expressed pH2B-eGFP ubiquitously, all Model I transgenic pigs developed neurological clinical signs starting at 4–5 months of age. All animals were unresponsive to treatment (antibiotics and steroids). Post mortem analysis identified severe multifocal Wallerian degeneration of brain and spinal cord segments (Fig 11) and generalized coagulopathy, with absence of necrosis or evidences of sepsis. The disseminated fibrin deposition in body cavities was likely related to the multifocal neuron degeneration, resulting in release of phospholipids in the blood stream, which has been associated with initiation of coagulation and coagulopathy disorders in human suffering from traumatic brain injury [68]. Wallerian degeneration is a process characterized by axonal degeneration due to nerve fiber discontinuity as a result of trauma/injury or impaired axonal transport [69, 70]. While this is by no means a comprehensive analysis of the complex neurological phenotype of Model I pigs, it does suggest that in two independent lines overexpression of pH2B-eGFP leads to axonal degeneration. We confirmed that the two lines have independent insertion sites suggesting this is not an insertional effect (Fig 2). We also demonstrated, via multiple methods, that cells from Model I animals express approximately twice the level of pH2B-eGFP than Model II cells (Fig 10). In addition, others have shown that in a direct comparison of the expression levels of the ACTB and the CAG promoter, the CAG promoter is approximately 10X more active that the ACTB promoter [71]. While our analysis only shows a 2X increase it is clear from our own data and previous reports that there are significant differences in expression between the two reporters. Interestingly, Brunneti and colleagues [71] generated transgenic pigs expressing cytoplasmic GFP using the a similar method described here (pCAG promoter/5’ flanking MARs/SCNT) and did not report any abnormalities in the established lines. We can only speculate that it is the fusion of the GFP with the pH2B histone that is leading to the phenotype observed. Thus, further experiments would be required to investigate why overexpression of pH2B-GFP leads to Wallerian degeneration. However, protein missfolding, aberrant degradation pathways, abnormal axonal transport, and/or protein accumulation are seen in many neurodegenerative diseases [72] so it is likely that excess levels of pH2B-GFP are triggering apoptosis of the neurons by one of the above mentioned mechanisms.

In conclusion, we describe here a viable pH2B-eGFP pig model generated using CRISPR-Cas9 driven homologous recombination system. This large animal model represents a valuable tool in the field of translational research, with multiple potential applications in the fields of stem cell transplantation, cell tracking/lineage fate studies, and for studying chromosome/histone 2B dynamics in different somatic cell types and during early embryogenesis of reconstructed SCNT embryos.

## Supporting Information

S1 Fig IRES-pH2B-eGFP CRISPR-Cas9 mediated homology directed repair (HDR) into the pig β-actin locus. Fluorescence-activated cell sorting of porcine fetal fibroblast 4 days post co-transfection with CRISPR-Cas9 and targeting vector. A 2.4% GFP positive population was sorted for generation of single cell colonies. Non transfected cells served as negative control, cells transfected with pMAX(GFP) served as positive control.
(PDF)

S1 Table Summary of pregnancies and outcomes from Somatic cell nuclear transfer (SCNT) and in vitro fertilization (IVF) of both Model I and Model II lines. (DOCX)

S1 File Nucleotide sequence of genomic insertion site for Model I lines. Sequence of flanking genomic region and the predicted genome mapping insertion site of the transgene for both Model I lines.
(DOCX)
