# Supplementary material for: Generation of a Stable Transgenic Swine Model Expressing a Porcine Histone 2B-eGFP Fusion Protein for Cell Tracking and Chromosome Dynamics Studies
Source: PLoS One. 2017 Jan 12;12(1):e0169242. doi: 10.1371/journal.pone.0169242 (PMC5230777; doi:10.1371/journal.pone.0169242)
Supplement: S1 Fig — Fluorescence-activated cell sorting of porcine fetal fibroblast 4 days post co-transfection with CRISPR-Cas9 and targeting vector. A 2.4% GFP positive population was sorted for generation of single cell colonies. Non transfected cells served as negative control, cells transfected with pMAX(GFP) served as positive control. (PDF) [file pone.0169242.s001.pdf]

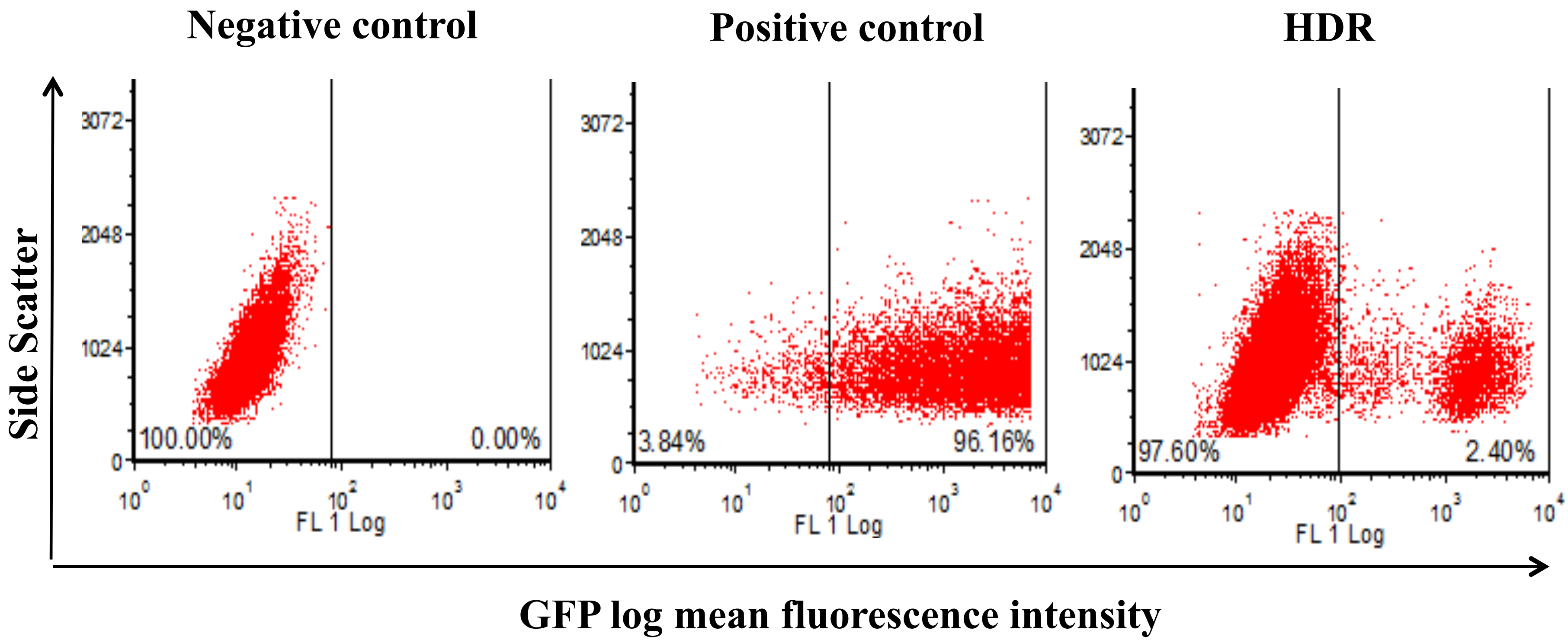


**S1 Figure. IRES-pH2B-eGFP CRISPR-Cas9 mediated homology directed repair (HDR) into the pig β-actin locus.**
