# Supplementary material for: Generation of a Stable Transgenic Swine Model Expressing a Porcine Histone 2B-eGFP Fusion Protein for Cell Tracking and Chromosome Dynamics Studies
Source: PLoS One. 2017 Jan 12;12(1):e0169242. doi: 10.1371/journal.pone.0169242 (PMC5230777; doi:10.1371/journal.pone.0169242)
Supplement: S1 Table — (DOCX) [file pone.0169242.s002.docx]

S1 Table. Summary of pregnancies and outcomes from Somatic cell nuclear transfer (SCNT) and in vitro fertilization (IVF).

| Pregnancy | Type of embryos | No. of transferred embryos | No. of piglets born^1^ | Efficiency (%) |
| --- | --- | --- | --- | --- |
| 1 | SCNT (Model I line 1) | 181 | 6/6 | 3.3 |
| 2 | SCNT (Model I line 2) | 126 | 7/7 | 5.6 |
| 3 | SCNT (Model II) | 119 | 3/3 | 2.5 |
| 4 | IVF (Model I line 1) | 212 | 2/3 | 1.4 |
| 5 | Model II AI | N/A | 10/15 | N/A |
| 6 | Model II AI | N/A | 3/6 | N/A |
| 7* | Model II AI | N/A | 6/13 | N/A |

SCNT = Somatic cell nuclear transfer, IVF = in vitro fertilization, AI = artificial insemination

^1^ Numbers are indicated as transgenic piglets/total number of piglets.

N/A = not applicable

* Pregnancy terminated at day 42 for fetal tissue collection
