# Supplementary material for: Generation of a Stable Transgenic Swine Model Expressing a Porcine Histone 2B-eGFP Fusion Protein for Cell Tracking and Chromosome Dynamics Studies
Source: PLoS One. 2017 Jan 12;12(1):e0169242. doi: 10.1371/journal.pone.0169242 (PMC5230777; doi:10.1371/journal.pone.0169242)
Supplement: S1 File — Sequence of flanking genomic region and the predicted genome mapping insertion site of the transgene for both Model I lines. (DOCX) [file pone.0169242.s003.docx]

**S1 File. Nucleotide sequence of genomic insertion site for Model I lines.**  Sequence of flanking genomic region and the predicted genome mapping insertion site of the transgene for both Model I lines.

**GenomeWalker insert sequence Model I line 1**

>GenomeWalker flanking sequence

CTTGTAGTTTTCAGAGTACAGGTCTTATGTTTCTTTAGGTAGGTTTACTCCTAGGTATTTTATTCTTTTGGATGGTGCAGTAAACAGGATTGCTTCCCTAATTTCTCTTTCTGATCTTTCATTGTTAGTGTATAGAAATGCAGTCGATTTCTGTGCCTTAACTTTGAATCCTGTGACTTTGCCAAATTCATGGATGACCTCTAACAGTTTTCTGGTAGAGTCTTTAGGATTCTCTAGGTATAGTATCATATCATCTCCAGTGATAGTTTTACTTCTTCCTTTCCAATTTGGATTCCTTTTATTTCTTTTACTCCTCTGATTTCTTTTACTCCTCTGATTGCTGCTAAGAATTTGTCCATTTCTTCTAGGTTTTCCATTTTATTGGCGTATAGTTGCATATAGTAGTCTCTTATGATCCTTTGTATTTTGGTGATGTCCATTGTTATTTCTCCTTTTTCATTTCTAATTTTATTGATTTTAGGACTTCCAAAACTATGTTGAAGTGTAGTGGCAAGAGCGGACATCCTTGTCTTGTTCCTGATCTCAGTGGGAATTCTTTCAGCTTTTCACCATTGAGAATGATATTCTCTGTGGGTTTCTTATATATGGCCTTTATGGTGTTGATGTAGCTTTCCTCTATGCCCACTTTCTGAAGGGTTTTTATCAGAAATGTGTGTTGGATTGTGTCAAATGCTTTCTCCGAGTCTATTGAGAGGATCATGTGGTTTTTATTCTTTAGTTTGTTAATGTGGTGTATCACAATGATTGATTTGCGAATATTGAAGAACTCTTGCATCTCTGGGATAAATCCTACTTGATCAAGATGTACAATCCTTTTAATGTATTTTGAATGAGGTTTGCTAATATTTTGTTGAGGATTTTTGCATTGAGTCATAANAAATTTTTCTCTACACTGAAGTCTTGATGGCATGCTTCTATATTATTTTCTAAAAGATTTAAAGTTTTGCCTTCTCCATTTAGACTTATAATTCACTGGAATTTTTTTGTGTGTATGGTATGACATATGGGTTCCCTTTTATTTTTTACATATAAATATATTTCCCTGTTTTTCTAAAAAAGAAAAAGACCATCATTTTCCCATTGTAAAATGCCATATTTTTTTCATAGGTCACTTACATATATCAATGGGCCTGTTTCTGAGCTCTACTCTATTTTA

Mapped to <http://useast.ensembl.org/Sus_scrofa/Location/View?db=core;r=1:25368175-25427711;tl=l8Jow023VfD7h62x-1889388-483094307>

**Whole Genome flanking Sequence Model I Line 2**

**Identified sequence**

> Scaffold GL896235 adjacent to human interferon-β matrix attachment regions

AGGGTCCTGGGGTGGGCGAGGCCAGGCCGTCCTGGCAGAGGAACAGCTCTGCGCAGGTCGGACACCCTTGCTGCCCCGTCCCTGTCAGTGATGCCAGGCCCGCTCACTGTCACCTGAGGGAGGGCCGCAGGTCTAAGCCAGCCATGCCACCCGCTGCCCTGTCCCTCTGCCCCCGAGGCCTCCTCCCAGAACCACTGGCCGCG

**Mapped to unassigned GL896235.1. Insertion at 400 bp location of sequence below**

>GL896235.1:14334-15134|insert_at_400bp_of_this_fragment

GTGCATGCTCCCGTTGGCCACTCACAGCTGGCACTTTGTGTCCTCAGCTATTGCCTCTGGAGCCTCGTCCTTTGACTTCAGGGGTTCCCCATGTGCCCGGTGGCGTTGTGGTCCACTGTGGGCTTGGGGGACAGTGACCAGACAGAGCTTGGGGTGTGCGTTTCGCCCAACAGAGCCTGCAAGCCAGGAGGGAGCCGCGGCCAGTGGTTCTGGGAGGAGGCCTCGGGGGCAGAGGGACAGGGCAGCGGGTGGCATGGCTGGCTTAGACCTGCGGCCCTCCCTCAGGTGACAGTGAGCGGGCCTGGCATCACTGACAGGGACGGGGCAGCAAGGGTGTCCGACCTGCGCAGAGCTGTTCCTCTGCCAGGACGGCCTGGCCTCGCCCACCCCAGGACCCTGGTGCCGGCAGAGGGGCTTTCAGCGAGATCCGCTGGTGACTGGATGTGTGGTGGGCGGCAGAGACGGGTGGGGTGNNNNNNNNNNNNNNNNNNNNNNNNNNNNNNNNNNNNNNNNNNNNNNNNNNNNNNNNNNNNNNNNNNNNNNNNNNNNNNNNNNNNNNNNNNNNNNNNNNNNGCAGAGGAGAGAGTCAGGGAGGCAGAGGGTCAGATGCCTCTCAGACACCCACCGGGAGGCGCTCAGAAGGGCCCTAGAGCAGGAGTGTCGGCCCTCCTGGTGGGAGAACCCACGGGGGGCAGAGGGGGGAATGAGTGCCGGGGGCGCCTCAGGGGAGGTGACCTGGGGCCAGAAACGAGGATGAGGGTGACCCAGGGCAGGAGGAGGCTGTGCAAAGGCCCTGAGGC
